# Characterization of Individuals with Sacroiliac Joint Bridging in a Skeletal Population: Analysis of Degenerative Changes in Spinal Vertebrae

**DOI:** 10.1155/2014/879645

**Published:** 2014-09-08

**Authors:** Takeshi Imamura, Kazunobu Saiki, Keishi Okamoto, Junichiro Maeda, Hiroaki Matsuo, Tetsuaki Wakebe, Keiko Ogami, Yoshitaka Manabe, Hironobu Koseki, Masato Tomita, Atsushi Tagami, Makoto Osaki, Hiroyuki Shindo, Toshiyuki Tsurumoto

**Affiliations:** ^1^Department of Macroscopic Anatomy, Graduate School of Biomedical Science, Nagasaki University, Nagasaki 852-8523, Japan; ^2^Department of Orthopaedic Surgery, Graduate School of Biomedical Science, Nagasaki University, Nagasaki 852-8523, Japan; ^3^Department of Oral Anatomy and Dental Anthropology, Graduate School of Biomedical Science, Nagasaki University, Nagasaki 852-8523, Japan

## Abstract

The aim of this study was to characterize the individuals with sacroiliac joint bridging (SIB) by analyzing the degenerative changes in their whole vertebral column and comparing them with the controls. A total of 291 modern Japanese male skeletons, with an average age at death of 60.8 years, were examined macroscopically. They were divided into two groups: individuals with SIB and those without bridging (Non-SIB). The degenerative changes in their whole vertebral column were evaluated, and marginal osteophyte scores (MOS) of the vertebral bodies and degenerative joint scores in zygapophyseal joints were calculated. SIB was recognized in 30 individuals from a total of 291 males (10.3%). The average of age at death in SIB group was significantly higher than that in Non-SIB group. The values of MOS in the thoracic spines, particularly in the anterior part of the vertebral bodies, were consecutively higher in SIB group than in Non-SIB group. Incidence of fused vertebral bodies intervertebral levels was obviously higher in SIB group than in Non-SIB group. SIB and marginal osteophyte formation in vertebral bodies could coexist in a skeletal population of men. Some systemic factors might act on these degenerative changes simultaneously both in sacroiliac joint and in vertebral column.

## 1. Introduction

Low back pains disturb daily activities to varying degrees in people throughout history. Degenerative changes not only in the lumbar spine but also in sacroiliac joint (SIJ) are involved in these pathological conditions—those in SIJ account for approximately 16% to 30% of cases of chronic low back pain [1–3]. Patients with an anterosuperior osteophytic bone bridge of SIJ were reported to have lumbar back pain [4]. Sacroiliac joint osteophytes cause sciatica for SIJ impinging on the sciatic nerve [5]. Clinically, they exist in individuals at a constant rate whose SIJ are united with bony bridges. Prevalence of sacroiliac joint bridging (SIB) was higher in males than in females and increased with ageing [6–8]. The degenerative changes in the SIJ are suspected as the pathogenesis of low back pains to some degree, and these pathological conditions progress to bony bridging of SIJ eventually. Martin et al. [9] reported a case of a patient with the symptom of secondary to anterior bridging of the SIJ; his pain was relieved by the surgical removal of the bony bridge across the anterior portion of the right SIJ. Moreover, patients with low back pain may be treated with the stabilization of the SIJ by means of noninvasive interventions [10, 11] or surgical techniques [12, 13]. Particularly, SIJ fixation operations using minimally invasive techniques have been reported with good outcomes [14, 15].

Meanwhile, the relevance of arthritis in SIJ and spondyloarthropathy is a cause of concern. Generally, the early symptom in a patient with ankylosing spondylitis (AS) can be varying degrees of low back pain; AS most commonly occurs in young males as persistent low back pain and stiffness that is worse in the morning and at night and improves with activity [16]. Recently a new disease concept for axial spondyloarthritis (axSpA), early stage of SIJ arthritis without radiological evidence, has been alerted [17–21].

We have already indicated that some general and systemic factors could work to affect osteoarthritis onset and progression in upper and lower extremities [22]. Furthermore, we hypothesized that the vertebrate bones in the individuals with SIB might have a general tendency to be highly ossified. Therefore, in this study, we evaluated the prevalence of SIB in a skeletal population and the degenerative changes in the whole vertebral column to characterize these individuals.

## 2. Materials and Methods

### 2.1. Materials

In this study, a total of 291 modern Japanese male skeletons were macroscopically examined. They were obtained from cadavers provided to Nagasaki University School of Medicine for anatomical dissection by medical students between the 1950s and the 1970s. They belonged to the same skeletal sample in our preceding study [9], and they were voluntarily donated nearly all by anonymous individuals. The present work does not pose any ethical problems from the viewpoint of the 2013 Declaration of Helsinki.

**Figure 1:**
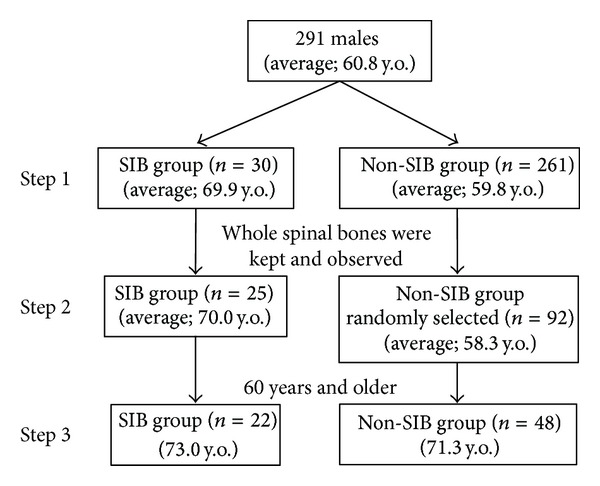
Materials of this study. A total of 291 skeletons were divided into SIB group and Non-SIB group (Step 1). Individuals whose whole spinal bones were kept and observed numbered 25 in SIB group and 92 in Non-SIB group (randomly selected) (Step 2). To focus on the degenerative changes with ageing phenomena in the vertebral bones, the statistical examination objects were confined to the skeletons older than 60 years old, 22 individuals from the SIB group and 48 individuals from the Non-SIB group (Step 3).

After they had been dissected, their soft tissues were removed to produce dry skeletal preparations. The sex and ages at death of all the individuals were registered. The mean age at death was 60.8 years, with a range of 19 to 89 years. They were divided into two groups: individuals with sacroiliac joint bridging (SIB group) and those without bridging (Non-SIB group) (Step 1 in Figure 1). To reveal the characteristics of these skeletons in the SIB group, about one hundred skeletons were selected randomly from the Non-SIB group for some statistical analyses (*n* = 92) (Step 2 in Figure 1). The ratios of the vertebrae bones which could be evaluated without any defects were 97.8% in the cervical vertebrae, 99.5% in the thoracic vertebrae, and 98.1% in the lumbar vertebrae; for nearly all of these spinal bones, almost all of the vertebral bodies and the zygapophyseal joints were evaluated. To focus on the degenerative changes with ageing phenomena in the vertebral bones, the statistical examination objects were confined to the skeletons older than 60 years old, 22 individuals from the SIB group (73.0 years old on average) and 48 individuals from the Non-SIB group (71.3 years old on average) (Step 3 in Figure 1). There was no significant difference in age between these two groups.

### 2.2. Sacroiliac Joint Bridging

**Figure 2:**
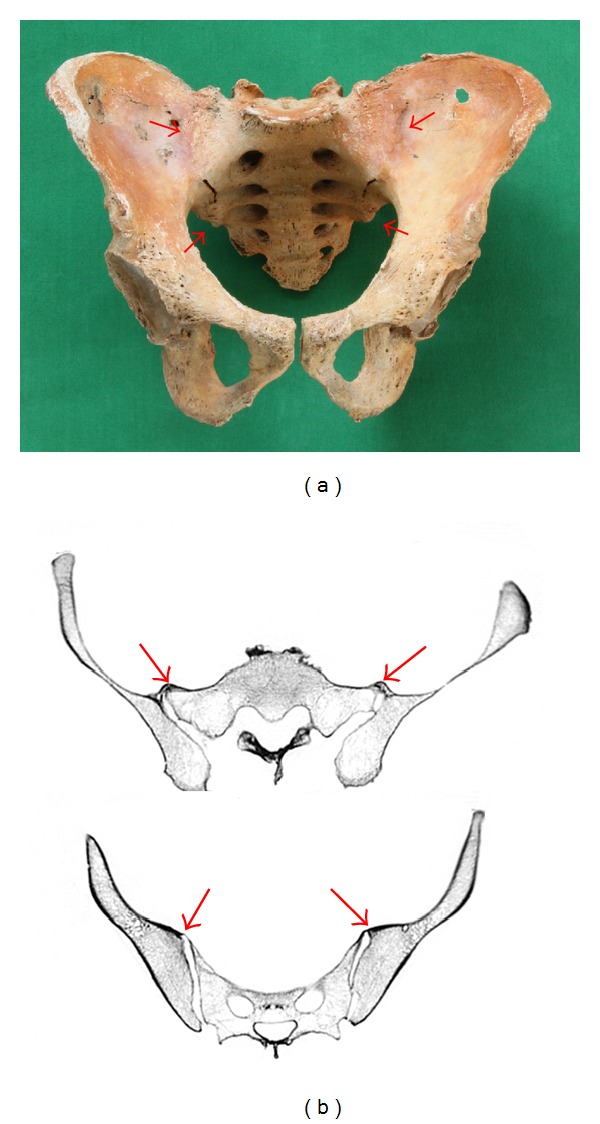
(a) Pelvic bone of a 79-year-old male; both sacroiliac joints are fused with bony bridging. The sacroiliac joints of both sides are unioned in upper and lower portions (indicated by red arrows). (b) CT images of the same pelvic bone: bony bridging is localized in the surface area (indicated by red arrows).

For each individual, the left and right SIJ were visually examined to categorize them into two groups: SIB group (Figure 2(a)) and Non-SIB group in Step 1. In the cases in which we were unable to assign group membership, computed tomography (CT) scanning images provided diagnostic clarity (Figure 2(b)). By contrasting these two groups, the marginal osteophytes around the vertebral bodies and degenerative changes of the zygapophyseal joints in the skeletons were selected randomly, evaluated, and characterized.

### 2.3. Marginal Osteophyte of Vertebral Bodies

**Figure 3:**
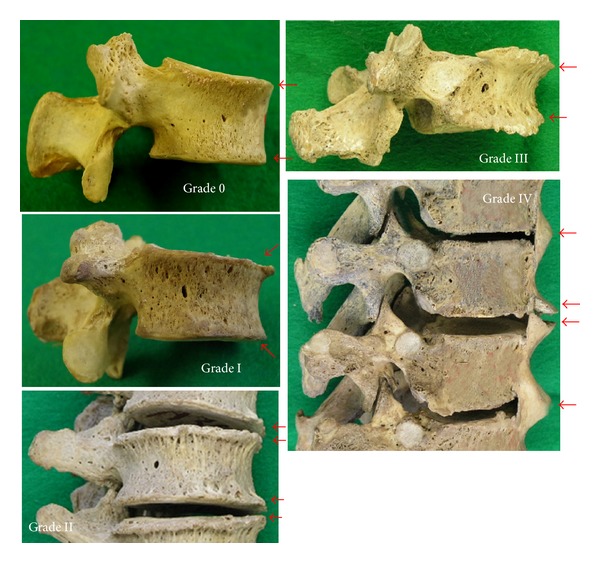
Marginal osteophytes of vertebral bodies (indicated by red arrows). Grade 0: the normal situation; Grade I: horizontally grown osteophytes; Grade II: vertically grown osteophytes; Grade III: significantly grown osteophytes; Grade IV: bridging osteophytes with adjacent vertebrae.

Marginal osteophytes of vertebral bodies were evaluated according to the diagnostic criteria reported earlier [23, 24] in Step 2 and Step 3: Grade 0: normal (no pathological changes), Grade I: horizontally grown osteophytes, Grade II: vertically grown osteophytes, Grade III: significantly grown osteophytes, and Grade IV: bridging osteophytes to adjacent vertebrae (Figure 3). The bones were scored at eight separate locations, including inferoanterior, inferoright, inferoposterior, and inferoleft segments of the upper vertebral body and superoanterior, superoright, superoposterior, and superoleft segments of the lower vertebral body. Then, the marginal osteophyte score (MOS) of each intervertebral space was calculated by averaging the total of the grade scores of the eight positions. Furthermore, the cases that contained more than one Grade IV area were defined as fused vertebra.

### 2.4. Degenerative Changes of Zygapophyseal Joints

**Figure 4:**
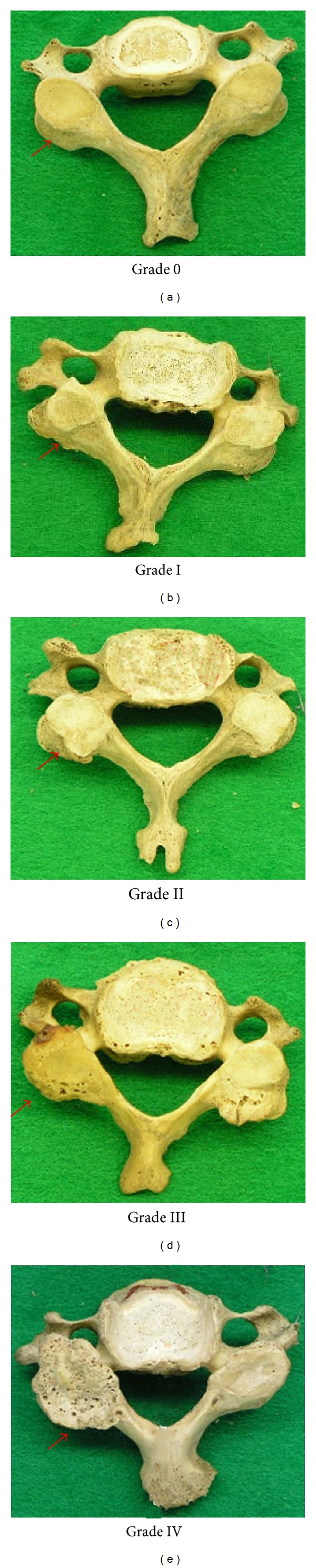
Degenerative changes of zygapophyseal joints (indicated by red arrows). Grade 0: the normal condition. Grade I: osteophytes grew on the rim of articular surface without pitting on the surface; Grade II: osteophytes grew on the rim of articular surface with lipping with slight pitting; Grade III: osteophytes grew all around the rim of the articular surface with moderate pitting on the surface and the rims of articular surface; Grade IV: osteophytes grew on the rim of articular surface with severe pitting on the surface.

Degenerative changes of the zygapophyseal joints, from the articulation of C2/3 to L5/S1 inclusive, were evaluated with the criteria reported earlier [25] in Step 2 and Step 3: Grade 0: normal (no pathological changes), Grade I: osteophytes on the rim of articular surface without pitting on the surface, Grade II: osteophytes on the rim of articular surface with lipping with slight pitting, Grade III: osteophytes all around the rim of the articular surface with moderate pitting on the surface and the rims of articular surface which tend to be broken, and Grade IV: osteophytes on the rim of articular surface with severe pitting on the surface and the rim becomes unclear (Figure 4). The grades were independently recorded for the right and left sides of the superior and inferior articular processes of the respective vertebrae. Then, the degenerative joint score (DJS) value for each intervertebral level was calculated by averaging the total of the eight grade numbers.

### 2.5. Statistical Analysis

The values of the correlation coefficient between MOS and the age at death and those between DJS and the age at death were tested in the SIB group and in the Non-SIB group. After adjustment for the age differences between these two groups as described in the results, these scores were statistically tested using the Wilcoxon test.

## 3. Results

### 3.1. Prevalence of SIB

Sacroiliac joint bridging was documented in 10.3% (30/291 male individuals) and 15.0% (26/148 male individuals) in people aged 60 years or older. In most of the pelvises in the SIB group, the anterior sacroiliac ligaments were ossified to varying degrees. In some cases, nearly the total area of the ligament was completely ossified. Moreover, in the other individuals, the ossification of the ligament could not be confirmed; instead, bony unions were recognized between both joint surfaces. These cases were examined closely with CT scanning. The average age of the 25 skeletons whose whole vertebral columns were curated in SIB group was 70.0 (range = 32–89) years. That was significantly higher than that of the 92 in the Non-SIB group at 58.3 (range = 19–83) years (*P* < 0.01).

### 3.2. Marginal Osteophyte Scores (MOS)

**Figure 5:**
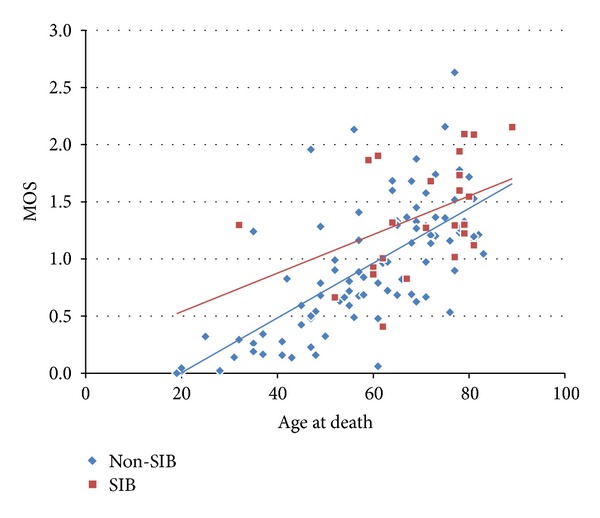
The relationship between the average values of marginal osteophyte scores (MOS) in all of the intervertebral spaces in respective individuals and their age at death. The correlation coefficient in Non-SIB group (blue diamond) was 0.69 (*P* < 0.01), and that in SIB group (red box) was 0.43 (*P* = 0.034).

The MOS values were calculated in respective intervertebral spaces from C2/3 articulation (between the 2nd cervical vertebra and the 3rd) to L5/S articulation (between the 5th lumbar vertebra and the bony sacrum). MOS in all of the intervertebral spaces in affected individuals were related to their age at death both in SIB group (*r* = 0.44, *P* = 0.033) and in Non-SIB group (*r* = 0.70, *P* < 0.01) (Figure 5).

**Figure 6:**
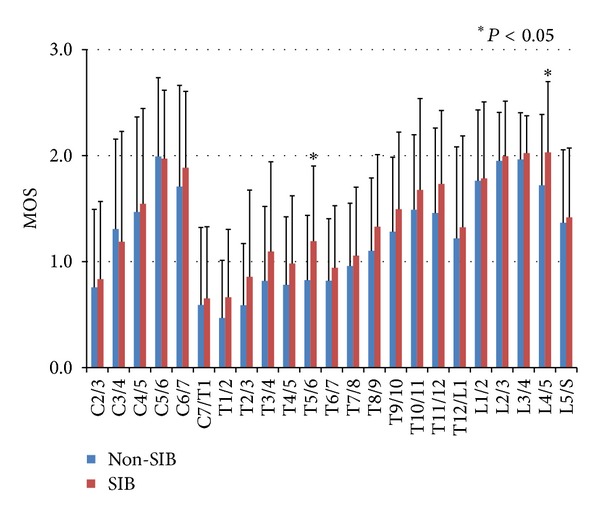
The averages and standard deviations of marginal osteophyte scores (MOS) on the intervertebral levels in 22 skeletons from SIB group (red) and 48 skeletons from Non-SIB group (blue). The bars indicate the standard deviations (S.D.). There was a significant difference between the two groups in only T5/6 level and L4/5 level, and the scores in the thoracic spines were consecutively higher in the SIB group than in the Non-SIB group.

Figure 6 indicates the average values of MOS on the intervertebral levels in 22 skeletons from the SIB group and in 48 skeletons from the Non-SIB group. Both of these two groups showed a similar pattern in MOS on the vertebral body. There were two large distributional peaks with the lowest peak at C6/7 or T1/2: the first peak at C5/6 and the second peak at L3/4 or L 4/5. Also, there were smaller peaks at T12/L1 in both groups. There was little difference between the values of the MOS of the two groups in cervical spines and lumbar spines. However, there was a significant difference between the two groups in T5/6 level (*P* = 0.030) and L4/5 level (*P* = 0.038); moreover, the scores in the thoracic spines were consecutively higher in the SIB group than in the Non-SIB group.

**Figure 7:**
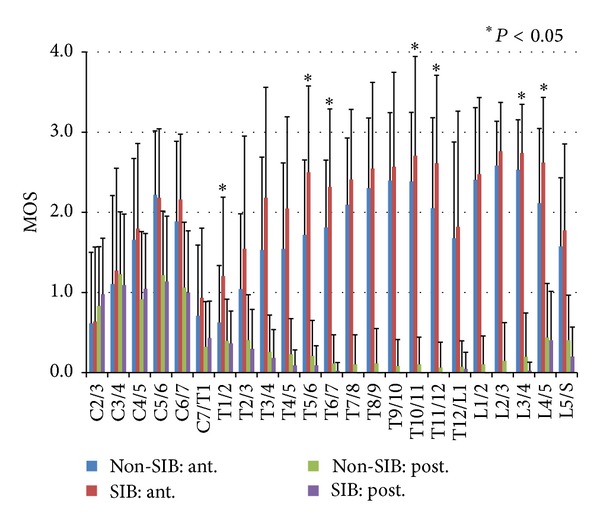
The averages and standard deviations of marginal osteophyte scores (MOS) on the intervertebral levels in all the same skeletons in Figure 6. Anterior part in Non-SIB group (blue) and SIB group (red) and posterior part in Non-SIB group (green) and SIB group (purple). The bars indicate S.D. Osteophyte formation was more dominant in the anterior part than in the posterior part, and, in the anterior part, the difference between both groups was prominent.

Figure 7 shows the average values of the MOS by comparing the anterior part and the posterior part of the vertebral bodies in both groups. Osteophyte formation was more dominant in the anterior aspect of the bone than in the posterior aspect. Particularly in the anterior aspect, the difference between both groups was proved to be significant; the average values of the SIB group were significantly higher than these of the Non-SIB group in T1/2 (*P* = 0.016), T5/6 (*P* < 0.01), T6/7 (*P* = 0.015), T10/11 (*P* = 0.049), T11/12 (*P* = 0.044), L3/4 (*P* = 0.043), and L4/5 (*P* = 0.025).

**Figure 8:**
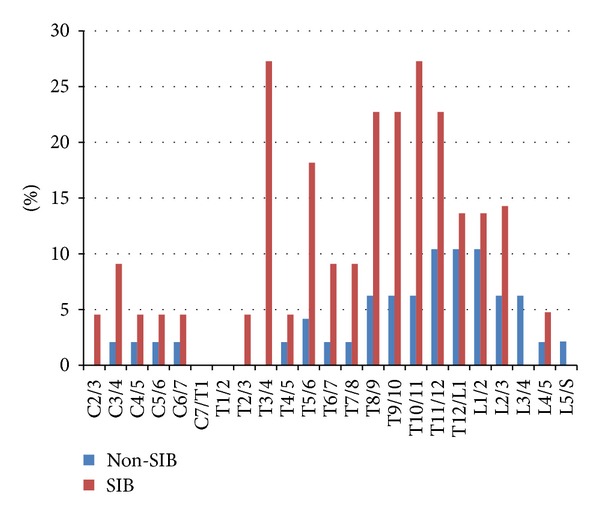
Percentage values of fused individuals in respective intervertebral spaces: Non-SIB group (blue) and SIB group (red). These values were obviously larger in the SIB group.

The values of percentage of the fused vertebral bodies in each intervertebral level were compared between both groups. These values were obviously larger in the SIB group (Figure 8). The number of fused vertebral bodies was significantly different. The average value per one skeleton was 2.41 in the SIB group, which was significantly higher than the value of 0.85 in the Non-SIB group (*P* < 0.01).

### 3.3. Degenerative Joint Score (DJS) in Zygapophyseal Joint

**Figure 9:**
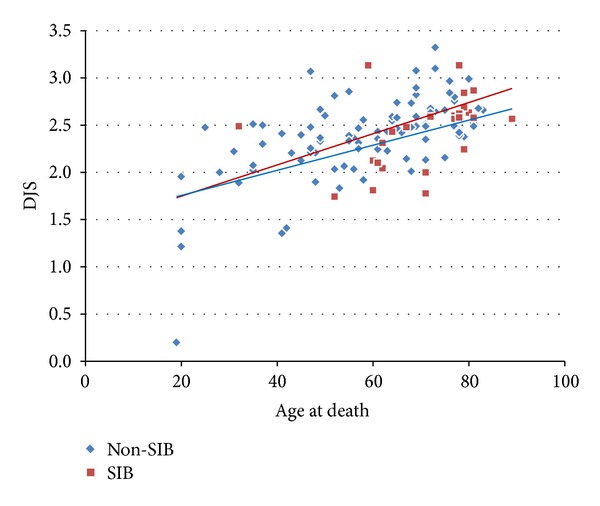
The relationship between the average values of degenerative joint score (DJS) of zygapophyseal joint in the whole intervertebral spaces of respective individuals and their age at death. The correlation coefficient in Non-SIB group (blue diamond) was 0.59 (*P* < 0.01), and that in SIB group (red box) was 0.39 (*P* = 0.54).

The relationship between the average values of DJS in all of the intervertebral spaces in respective individuals and their age at death was shown in the scatter chart (Figure 9); the correlation coefficient in the Non-SIB group was 0.62 (*P* < 0.01), and that in the SIB group was 0.39 (*P* = 0.054).

**Figure 10:**
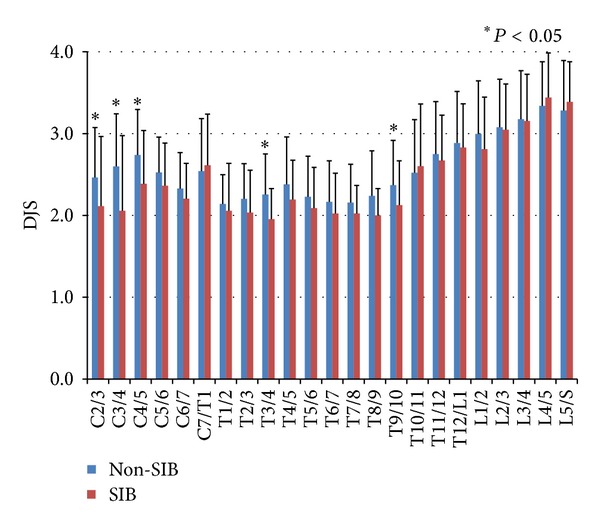
The averages and standard deviations of degenerative joint score (DJS) of zygapophyseal joint in the intervertebral spaces in 48 skeletons from Non-SIB group (blue) and 22 skeletons from SIB group (red). Average scores of DJS increased gradually from C2/3 to the lower cervical level, and they kept almost flat in the thoracic vertebrae. However, they increased again gradually from T9/10 to the peak of L4/5. The values of score in the Non-SIB group were higher than those in the SIB group from the cervical to the thoracic vertebrae consecutively.

As stated previously, the objects were confined to the skeletons which were 60 years and older. For 22 individuals in the SIB group and 48 in the Non-SIB group, the average values of DJS in respective intervertebral levels were calculated (Figure 10). Average scores of DJS increased gradually from C2/3 to the lower cervical level, and they kept almost flat in the thoracic vertebrae. However, they increased again gradually from T9/10 to the peak of L4/5. The values of score in the Non-SIB group were higher than those in the SIB group from the cervical to the thoracic vertebrae consecutively. In particular, the scores of the former were significantly higher in C2/3 (*P* = 0.033), C3/4 (*P* < 0.01), C4/5 (*P* = 0.031), T3/4 (*P* = 0.022), and T9/10 (*P* < 0.01). Little difference was recognized between both groups in the lower intervertebral levels than T9/10.

## 4. Discussion

It has been stated that osteophytes on the vertebral margin develop in an attempt to strengthen the vertebral bodies in response to continual pressure and weakening of the skeletal structure with ageing [26–29]. Therefore, in a normal spine, vertebral osteophytes do not develop before the vertebral epiphyseal rings have fused which occurs at around 20 years of age [26]. On the other hand, in the iliac articular facet, the first alterations of cartilage structure can be detected around the onset of puberty [30]. The SIJ can fuse not only with bone proliferating changes and the aging process, but also with diffuse idiopathic skeletal hyperostosis (DISH) and seronegative spondyloarthropathies such as ankylosing spondylitis (AS) or psoriatic arthritis. Resnick [31] stated that bony ankylosis in degenerative diseases resulted from para-articular bridging osteophytes, whereas the true intra-articular ankylosis characteristic of ankylosing spondylitis was generally absent. He observed that osteophytes were a feature of degenerative sacroiliac disease and were predominant on the anterior surface of the ilium and sacrum but were not prominent in ankylosing spondylitis. Moreover, Dar et al. [32] reported that SIB was dominant in the superior region of this joint. However, there is the possibility the current study may have included some skeletons of seronegative spondyloarthropathies such as ankylosing spondylitis, but it is unlikely to have had an effect on the results due to the fact that there is a lower prevalence of this disease in Japan (6.5 out of 100,000) [33].

Waldron and Rogers [34] investigated the skeletons of the 18th and 19th centuries from a crypt in England, and they reported that the prevalence rates of sacroiliac fusion were 6.3% in the males and 4.3% in females. Dar et al. [32] analyzed 2845 skeletons from an American osteological collection of people who died during the first half of the 20th century and found that SIB was present in 12.27% of the males, contrasting with only 1.83% of the females; these changes were independent of ethnic origin but were age dependent. In our study, bridging was present in 10.3% of Japanese males. This is the first report on the frequency rate of SIB in Asian people.

Excessive mechanical stress, particularly at a younger age, may predispose one to osteophyte formation later in life [35, 36]. Watanabe and Terazawa [37] stated that the marginal osteophyte on the vertebral bodies began to form around the age of 30 in both sexes. Differences between individuals owed to a response to erect posture during bipedal locomotion rather than differences in occupational stress [38]. In this study, osteophytes were not present until the age of around thirty either; and osteophytes increased with aging, which was significantly correlated with age. In the cervical vertebrae, several authors had a consensus of opinion: C5/6 and C6/7 have the greatest frequency of osteophyte formation, attributed to their mobility and load-bearing nature [39–41], and O'Neill et al. [35] reported that 681 women and 499 men over 50 years of age had thoracic osteophytes most frequently on T9 and T10. Moreover, Van der Merwe et al. [42] investigated a total of 101 male and 117 female morphologically normal vertebral columns and found that the highest frequency and degree of projections were on C5, T11, T12, L3, L4, and L5, while the lowest frequency was observed on T2 and L1 in females and on T2 in males. Nathan [26] examined 346 white and black male and female individuals and observed that the highest incidence of osteophytes in each region was in the vicinity of the peaks of the spinal curves (C5, C8, and L3-4), whereas the lowest frequencies were found where the line of weight-bearing crosses the spine (T1, T12, and L5-S1). In this study, the higher osteophyte scores were on C5/6, L2/3, L3/4, and L4/5 and the lowest score was observed on T1/2; the thoracic region had less bony spur development than both the cervical and lumbar vertebrae. This might be because they are more stable due to the presence of the ribs and less mobile than the other vertebrae [43]. Under these situations, thoracic vertebrae, with limited influence of mechanical stress, might be sensitive to the individual's general tendency for additional bone forming. Moreover, it has been reported that vertebral deformity and osteoarthritis are frequent in osteoporotic vertebrae in the aged individuals [44]. In this study, as compared with the Non-SIB group, marginal osteophyte scores were higher in the SIB group, especially in thoracic vertebrae. Moreover, the frequency of vertebral body fusion was evidently higher than in the Non-SIB group. Considering these findings, it was suggested that there was a tendency to form bone with aging in the SIB group.

Master et al. [45] investigated the prevalence of combined lumbar and cervical arthrosis in a large population sample and examined its association with age, sex, and race. They confirmed that lumbar arthrosis and advancing age were associated with cervical arthrosis independent of race and sex, and they proposed that lumbar arthrosis and age were associated with cervical arthrosis. Just as Higuchi [25] reported, in this study, degenerative scores of zygapophyseal joint increased gradually from the upper cervices with the peak of C4/5, and they increased again in the lower thoracic vertebrae and lumbar vertebrae. The correlation coefficient between the scores and age at death in the Non-SIB group was significantly high, but correlation within the SIB group was not significant (*P* = 0.54); this meant that degenerative changes in zygapophyseal joints developed and proceeded with little correlation with the ageing process in the SIB group. Additionally, the values of degeneration scores of zygapophyseal joint in cervical and thoracic vertebrae were higher in the Non-SIB group than in the SIB group. Considering the result that, in the SIB group, the marginal osteophyte scores were higher and the frequency of vertebral body fusion was higher than in the Non-SIB group, it was possible that intervertebral mobility was restricted to reduce the mechanical loads on zygapophyseal joints. Apparently, this might be the reason why there were discrepancies between marginal osteophyte formation and zygapophyseal degeneration in the two groups.

Recently a new concept disease of axSpA, which was once understood as an early stage of AS, has not necessarily been interpreted as the same entity as AS by some researchers; this is because the gender ratios were different between axSpA and AS [20, 46, 47], and a number of studies demonstrate that AS and axSpA differ in their genetic property (HLA-B27 typing) [46]. This axSpA has far greater clinical heterogeneity and has a broader aetiopathogenesis; the natural history of axSpA has not yet been reliably established [46]. Considering these heterogeneities, some of the skeletons classified to the SIB group in this study could belong to some kinds of this entity.

New bone formation of the entheses with possible progression to ankylosis is among the hallmarks in these patients of axSpA [48]. TNF antagonists were effective to control inflammation in the long time in axSpA. However, once the bone formation process is underway, it may not be possible to slow the rate of new bone formation in axSpA [49]. According to another hypothesis, inflammation and new bone formation may be triggered by the same factor and then go on to develop independently of each other via different molecular mechanisms [48]. It may be possible that these hypotheses were suggestive of considering the etiology of the bone forming phenomena in the SIB group of this study.

Waldron and Rogers [34] investigated the modern skeletons of 41 individuals with the SIJ's fusion compared with 82 adult skeletons without that condition. This study showed a significant association between sacroiliac fusion and the presence of DISH and osteoarthritis of the spine, but not for osteoarthritis at any other site. Moreover, Dar et al. [50] studied 289 human male skeletons for the presence of SIB, entheseal ossification, cartilaginous calcification, and other axial skeleton joint fusions; they stated that SIB was strongly associated with entheseal reactions in other parts of the body. With our study, it was indicated that SIB and marginal osteophyte formation could coexist in vertebral bodies in a skeletal population of men. Therefore, it is likely that some combination of systemic factors, for example, genetic, nutritional, hereditary, or hormonal factors, might act on these degenerative changes simultaneously both in SIJ and in the vertebral column. These findings might indicate a new concept about the pathological conditions with systematic bone formation tendencies in the human axial skeletons. Further studies, including genetic analysis, might be required for establishing it.

## 5. Conclusions

Sacroiliac joint bridging and marginal osteophyte formation in vertebral bodies could coexist in a skeletal population of men. Some systemic factors might act on these degenerative changes simultaneously both in sacroiliac joint and in vertebral column.
